# The Book World of Medicine and Science

**Published:** 1894-11-10

**Authors:** 


### THE BOOK WORLD OF MEDICINE AND SCIENCE.

The Jewish Method of Slaughter Compared with other Methods from the Humanitarian, Hygienic, and Economic Points of View. By J. A. Dembo, M.D. Translated from the German with the Author's amendments. (London: Kegan Paul, Trench, Trübner and Co., 1894. pp. 111. 2s. 6d. net.)

This treatise sums up boldly in favour of the Jewish method. In regard to humanity everything hinges on the rapidity with which not death but unconsciousness supervenes; and this, the author says, happens within the lapse of three to five seconds. He speaks very strongly of the cruelty of the system of transfixion of the neck commonly adopted for killing sheep and calves, by which the blood escapes far more slowly than in the complete division of the arteries according to the Jewish method. As to the various stunning systems with the mallet, the pole-axe, &c., he asserts that it is quite impossible to produce immediate unconsciousness by a single blow with that certainty which alone would make it justifiable, and instances cases which he has seen in which five or more blows were necessary. Several drawings are given of the result of "neck-stab," a method of slaughter adopted in some places, showing that so far from touching the medulla the stab only divided the cord some distance lower down. If this is so it is clear that this procedure is far from being the sudden and painless extinction which it is claimed to be. Turning to the hygienic side of the question Dr. Dembo lays great stress on the advantage of leaving as little blood in the meat as possible both as regards the character of the meat and its lessened tendency to undergo putrefactive change. The book is an interesting *résumé* of many interesting facts regarding a subject which is not very attractive, and concerning which there is evidently a good deal of ignorance, and, as we have already said, it sums up strongly in favour of the Jewish method by complete division of the vessels of the neck.

The Insanity of Over-Exertion of the Brain. By J. Batey Tuke, M.D., F.R.C.P.E. (Oliver and Boyd, Edinburgh; Simpkin, Marshall, Hamilton, Kent, and Co. (Limited), London. Price 6s.)

This book consists of the Morison Lectures, delivered before the Royal College of Physians of Edinburgh in 1894, by Dr. Batey Tuke. In it he discusses the pathology and treatment of idiopathic insanity, or, as he prefers to call it, the insanity of over-exertion of the brain. These lectures occasionally recall former utterances by Dr. Tuke. He seems to think that some of his professional brethren still believe that the mind is a thing apart from the body, and here and there one has just a suspicion that he is frowning down upon them from a pinnacle of enlightened superiority. Recognizing the importance of an accurate knowledge of the minute anatomy of diseased organs, he gives a careful description of the structure of a cerebral convolution, and illustrates it by means of a coloured diagram. His idea is a good one, but it would have been more convenient had the explanatory notes been placed to the diagram and not 14 pages away. Evidences of wide and careful reading are to be found, and the chapters are bright and attractive. The author shows that moral causes act only through physical means, and that prolonged stimulation, followed by exhaustion of cell function, and attended by hyperæmia, may pass into inflammation with structural alterations. We quite agree with him that the influences of peripheral irritation in the production of insanity has been greatly exaggerated, and more especially so with regard to the female organs of generation. His chapter on treatment is most interesting. We are glad to see that he strongly insists upon the necessity of obtaining rest for the brain, and points out how important it is that the patient should be kept in bed during the acute stage. This method of treatment is of course open to abuse, but it is the rational one. The smallness of the numbers confined to bed in an asylum is not always a point to be mentioned in terms of commendation. We think also that the lecturer does not do justice to the present asylum nurse. It is as unfair to compare her with the "old attendant," as to compare the hospital nurse of to-day with the "Mother Gamp" of the past.

